# Subcellular and Dynamic Coordination between Src Activity and Cell Protrusion in Microenvironment

**DOI:** 10.1038/srep12963

**Published:** 2015-08-11

**Authors:** Yue Zhuo, Tongcheng Qian, Yiqian Wu, Jihye Seong, Ya Gong, Hongwei Ma, Yingxiao Wang, Shaoying Lu

**Affiliations:** 1 Department of Bioengineering, Beckman Institute for Advanced Science and Technology, University of Illinois at Urbana-Champaign, Urbana, IL 61801; 2 Neuroscience Program, Beckman Institute for Advanced Science and Technology, Department of Molecular and Integrative Physiology and University of Illinois at Urbana-Champaign, Urbana, IL 61801; 3 Center of Biophysics and Computational Biology, Beckman Institute for Advanced Science and Technology, University of Illinois at Urbana-Champaign, Urbana, IL 61801; 4 Department of Bioengineering, Institute of Engineering in Medicine, University of California, San Diego, La Jolla, CA 92093-0435; 5 Suzhou Institute of Nano-Tech and Nano-Bionics, Suzhou, China; 6 Center for Neuro-Medicine, Brain Science Institute, Korea Institute of Science and Technology (KIST), Seoul, South Korea

## Abstract

Migration of endothelial cells is essential for wound healing and angiogenesis. Src kinase activity plays important roles at the protrusions of migrating endothelial cells. However, the spatiotemporal coordination between Src kinase activity and the protrusion of cell edge remains unclear. Therefore, we investigate these coordinated molecular events at the initiation of cell migration, by integrating microfabrication, fluorescence resonance energy transfer (FRET)-based biosensors, and automated computational image analysis. We demonstrate that the physical release of restrictive micropattern triggered a significant decrease of Src activity at the protrusive edge of endothelial cells. Computational cross-correlation analysis reveals that the decrease of Src activity occurred earlier in time, and was well-coordinated with the protrusion of cell edge in polarized cells, but not in non-polarized cells. These results suggest that the spatiotemporal control of Src kinase activity is well-coordinated with cell polarization and protrusion in endothelial cells upon the release of physical constraint, as that experienced by endothelial cells sprouting from stiff tumor micro-environment during angiogenesis. Therefore, our integrative approach enabled the discovery of a new model where Src is de-activated in coordination with membrane protrusion, providing important insights into the regulation of endothelial migration and angiogenesis.

The migration of endothelial cells plays essential roles in embryogenesis, tissue regeneration, wound healing, and angiogenesis in cancer^1^,^2^,^3^,^4^. During tissue regeneration and angiogenesis, endothelial cells are programed to migrate toward and proliferate at the site of nascent blood vessels, which supply nutrients for the maintenance and growth of surrounding tissues^5^. Therefore, understanding the underlying mechanisms regulating endothelial cell migration has important implications in regenerative medicine and cancer therapeutics.

The initiation of cell migration is regulated by an integrative signaling network involving many functional molecules. It is generally believed that the activation of the tyrosine kinase Src^6^,^7^,^8^ and its downstream signaling molecules, including the small GTPase Rac1 and Arp2/3 complex, is required for the polymerization of branched actin meshwork and the initiation of membrane protrusion^9^,^10^,^11^. Src, Rac1 and PI3K have also been reported to form a positive feedback loop at the lamellipodia to promote cell protrusion and migration^12^,^13^.

Meanwhile, several lines of evidence suggest another small GTPase, RhoA, as a key player in the initiation of cell migration. RhoA has been shown to be activated closer and faster at the migration front than Rac1^14^. Since cell protrusion has been reported to occur before Rac1 activation^14^,^15^, it is possible that RhoA and its downstream effector mDia can trigger cell protrusion without Rac1^16^,^17^,^18^. Recent discoveries of unbranched and differentially oriented actin networks in lamellipodia also support this notion^19^,^20^. Because of the mutual inhibition between Rac1 and RhoA, Src and Rac1 activities may need to be transiently reduced at the cell edge to allow the initiation of protrusion and migration. In fact, it has been shown that Src activity involved in cell migration is differentially regulated at different subcellular locations^7^,^8^ while the overall role played by Src kinase in the initiation of cell migration remains unclear.

To investigate the spatiotemporal partition of Src activity at the protrusion front of endothelial cells, soft-lithography-based microfabrication, fluorescence resonance energy transfer (FRET)-based live cell imaging, and automated image analysis methods are integrated to stimulate cell migration, visualize and quantitatively analyze the intracellular molecular activity and its correlation with cell protrusion. Microfabrication has been widely applied in live cell imaging to mimic and provide a controllable micro-environment in extracellular matrix (ECM)^14^,^21^,^22^,^23^,^24^,^25^,^26^,^27^,^28^. In this work, a novel micropatterned PDMS gel membrane was designed to first constrain the movement of the cells and then release the cells to trigger protrusion, polarization and migration (Fig. 1A)^29^.

FRET-based biosensors provide powerful tools for visualizing dynamic changes of molecular activities in live cells^30^,^31^. Src FRET biosensors have been developed by our group and utilized to monitor the spatiotemporal dynamics of Src kinase activity in endothelial cells with high spatiotemporal resolutions^7^,^30^. Active Src kinases can cause tyrosine phosphorylation on the specific biosensor substrate, the intramolecular binding between substrate phosphor-tyrosine and Src Homology 2 (SH2) domain, and the subsequent increase of ECFP/FRET emission ratio (Fig. 1B). Therefore, the values of ECFP/FRET ratio represent the level of intracellular Src kinase activity. The ratiometric signal of the biosensor is independent of cellular expression level and subcellular transport. It is more accurate than the readout of intensity-based fluorescence proteins^31^. Our Src biosensors have been extensively characterized to confirm that the ratiometric biosensor signal is sensitive and specific to Src kinase activity *in vitro* and in cells^13^,^30^,^32^,^33^. The Src biosensors can be activated by growth factors in both endothelial and HeLa cells, but not in SFY–/– cells which lack Src. The activation can be inhibited by the specific Src inhibitor PP1^13^,^32^. Furthermore, the Src biosensor can be engineered to target at plasma membrane to efficient monitor local Src kinase activity. Therefore, we utilize a membrane-bound KRas-Src biosensor to monitor the dynamic activity of Src kinase at the plasma membrane in the cells stimulated by constraint release (Fig. 1B)^30^,^34^.

Automated and high throughput computational imaging analysis methods are essential for accurately analyzing the spatiotemporal cell membrane dynamics. Semi-automated contour-based analysis methods have been used to measure protrusion activity of the cell^35^,^36^,^37^,^38^,^39^,^40^. Recently, more sophisticated algorithms have been developed to track the protrusive movement of the cells during adhesion or random migration^41^,^42^. The level-set-based approach developed by Machacek *et al*. has been adopted to reconstruct the continuous motion of cell membrane based on video images^14^,^42^. However, it is not generally applicable to whole-cell tracking of membrane protrusions and computationally demanding. Here, we developed a whole-cell tracking level-set method with a balance between tracking accuracy and computing cost, for the quantification of membrane protrusion in live cells. As such, we can detect and track the movement of cell membrane for an extended period of time, as well as quantify and correlate the dynamic Src kinase activity indicated by FRET signals.

## Results

### The release of micropatterned gel membrane induced cell polarization, protrusion, and differential regulation of sub-cellular Src activity

**Figure 1:**
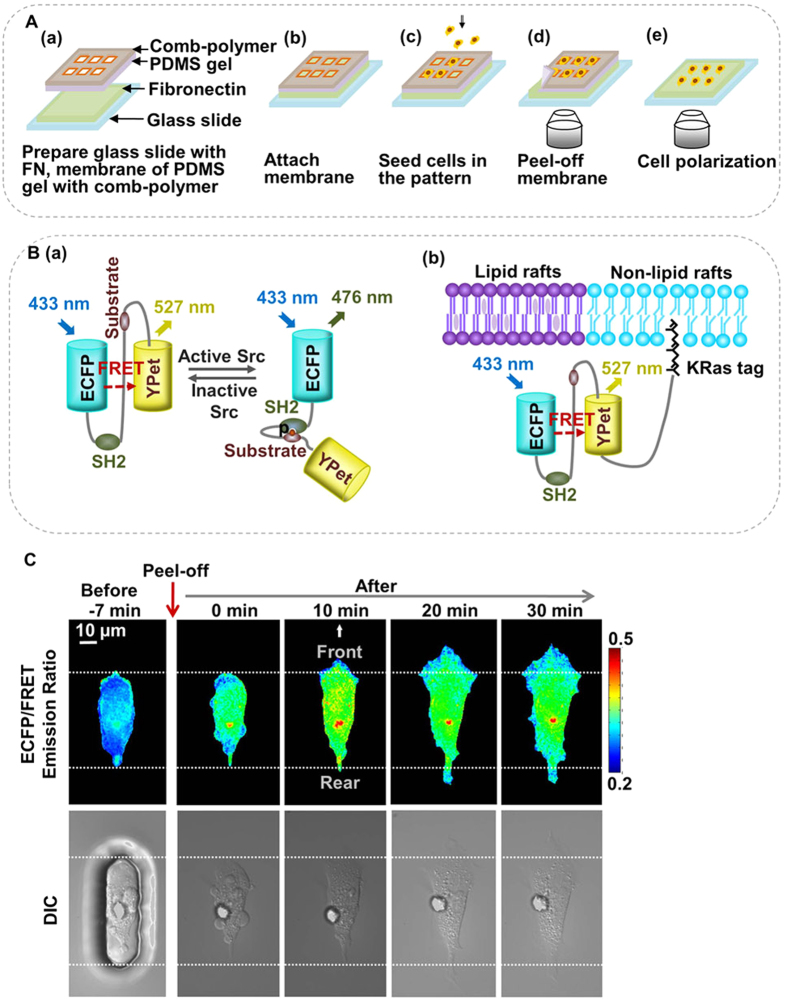
Src activity was down-regulated at the protrusion of a cell released from micropattern constrained space. (**A**) A flowchart of the experiment: (a) coat comb-polymer on the surface of a PDMS gel membrane, coat fibronectin (FN) on the surface of a cover glass slide; (b) attach the PDMS gel membrane to the glass slide; (c) seed cells inside the micro-patterned wells within the membrane, and start imaging; (d) peel-off the membrane during imaging; (e) observe the molecular activity during cell protrusion and polarization. (**B**) The schematic drawing of the Src biosensor, with its activation mechanism and membrane targeting strategy. (a) The biosensor contains an ECFP, a flexible linker connecting SH2 domain and a substrate sequence, and a YPet. The biosensor substrate can be specifically phosphorylated by active Src kinase, subsequently binding to the intramolecular SH2 domain, and causing a reversible increase of ECFP/FRET intensity ratio. (b) A KRas-tag was engineered to the C-terminal to insert the biosensor in the membrane microdomains outside of lipid rafts in live cells. (**C**) Visualization of ECFP/FRET emission ratio of Src biosensor at the initiation of constraint-released cell protrusion. Top panels show the ECFP/FRET emission ratio images; lower panels show the DIC images.

As shown in Fig. 1A, biosensor-expressing human umbilical vein endothelial cells (HUVECs) were initially seeded in the wells of micro-patterned gel membrane^29^. Peeling off the gel membrane released the cells from constraining, inducing cell protrusion and polarization. Src FRET biosensors allow the visualization of subcellular Src activity simultaneously with the protrusion dynamics at cell edge (Fig. 1B,C). Our results show that about half of the cells started to polarize and protrude in ~10 minutes after release (Fig. 1C). Meanwhile, the ECFP/FRET emission ratio of the Src biosensor changed significantly with distinct subcellular patterns, indicating differential regulation of Src activity in the cell upon release (Fig. 1C and Supplementary Video 1). Strikingly, Src activity turned significantly lower at the protrusive lamellipodial regions than at the non-protrusive regions 10–30 minutes after release. ECFP and FRET intensity images confirmed the correct localization of the biosensor at cell protrusions (Supplementary Fig. 1 and Supplementary Video 1). We hypothesize that the release of constraint caused the down-regulation of Src activity at the protrusive regions and focus on the quantitative analysis of this regulation for the rest of the manuscript. Meanwhile, the ECFP/FRET ratio image of the cell body changed color from cold to hot near the center of the cell body, indicating a significant increase in Src activity, probably promoted by the stabilized interaction between integrin receptors and ECM proteins^43^,^44^. Discrete spots of high ECFP/FRET ratio were also observed at the center of the cells, possibly indicating biosensor proteins trapped in perinuclear organelles due to the deficiency in secretion pathways (Fig. 1). This portion of the cells was not included into the quantitative analysis.

### Tracking the evolution of cell boundary and sampling subcellular Src activity

**Figure 2:**
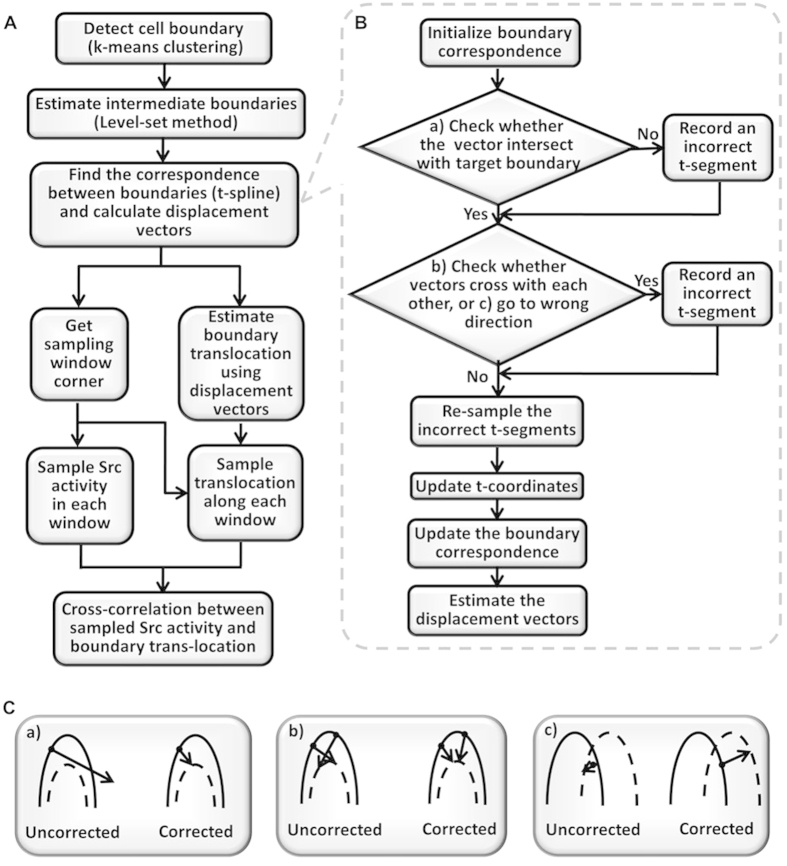
The imaging analysis procedure. (**A**) The flow chart of the overall image analysis procedure; (**B**) The flow chart for the calculation of displacement vectors; (**C**) Examples for correction of displacement vectors.

**Figure 3:**
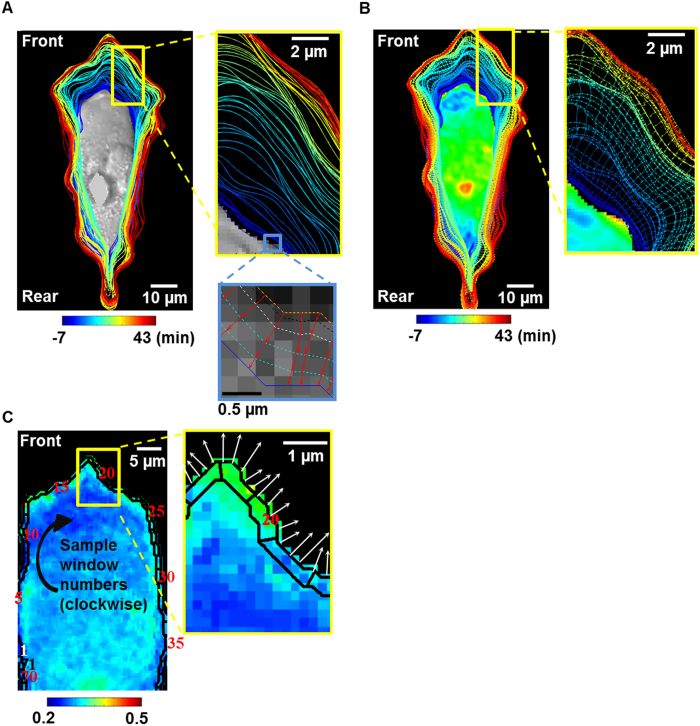
The boundary evolution and displacement results of a protruding cell. (**A**) Detected boundaries of a protruding cell is plotted with the intermediate boundaries estimated by the level set method, in solid lines color-coded by time (early-blue, late-red, right panels: close-up views); (**B**) Boundary of the cell plotted with displacement vectors connecting corresponding reference points, colored-coded by time (early-blue, late-red, right panel: a close-up view); (**C**) The ratio image of a protruding cell is overlaid with sampling windows (solid black lines). Right panel: the cells with sampling window is shown in a closer-up view with the estimated displacement vectors (white arrow).

To quantitatively evaluate the spatiotemporal maps of membrane protrusion and Src activity, we developed a level-set based algorithm for tracking the evolution of cell boundary and sampling subcellular Src activity along the boundary (Fig. 2). First, the cell boundary was detected using the k-means clustering algorithm^45^. Then, the temporal evolution of the cell boundary was propagated by the Level Set Method (LSM) to enhance temporal resolution and calculate the correspondence between two consecutive cell edges^14^,^42^. To track the evolution of the whole-cell boundary, the spline representation of the cell edge is used to resolve geometric conflictions, in the cases where a correspondence was not found between the reference points of two consecutive cell edges, two correspondence vectors intercept each other, or the correspondence is found toward another direction (Fig. 2B,C, Materials and Methods). The heuristic corrections successfully resolved the geometric conflictions without reducing the step-size of the LSM and sacrificing computing efficiency. The resulting boundary evolution and correspondence vectors for a representative cell are shown in Fig. 3.

Subsequently, the boundary correspondence was used to evolve the sampling windows where average Src ECFP/FRET ratio was quantified as well as the displacement vector for calculating boundary translocation (Fig. 3). The resulting displacement vectors and sampling windows are shown in Fig. 3B,C. This cell had about 71 sampling windows located at 0–139 μm from a reference point along the cell edge. Therefore, the average Src ECFP/FRET ratio and cell edge translocation could be evaluated in each sampling window as a function of time, and the Src-translocation coordination can be evaluated using the cross-correlation functions (Fig. 2A)^14^. Based on quantified membrane translocation, cell boundary can be characterized as protrusive and non-protrusive regions (Supplementary Fig. 2). Thus, the cells were classified into polarized and non-polarized groups, as detailed in the methods section (Supplementary Fig. 2). Polarized cells have a single protrusion region and a well-defined polarity with a protrusion front (Fig. 3). These definitions and classification allow the quantitative evaluation of the molecular regulations presented in the next sections.

### Quantitatively evaluate the spatiotemporal correlation between Src activity and edge protrusion in polarized cells

**Figure 4:**
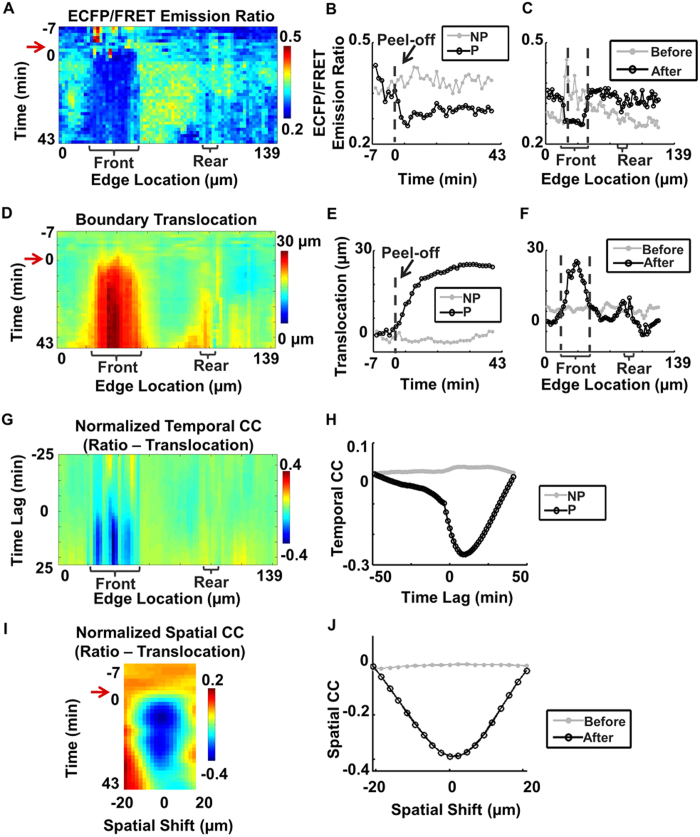
Quantify the dynamic Src activity and membrane protrusion at the edge of a polarized cell. (**A**) The spatiotemporal map of ECFP/FRET ratio at cell edge; (**B**) The time courses of Src FRET ratio in a representative sampling window at the protrusive edge (P) and non-protrusive edge (NP); (**C**) The line scans of Src activity along the cell edge before and after removing the gel membrane; (**D**) The spatiotemporal map of cell boundary translocation; (**E**) The time courses of edge translocation at the protrusive (P) and non-protrusive (NP) edges; (**F**) The line scans of translocation at the cell edge before and after removing the gel membrane; (**G**) The temporal cross-correlation (CC) map between Src activity and cell edge translocation; (**H**) temporal CC between Src activity and boundary translocation in two representative sampling windows (P-region and NP-region); (**I**) spatial CC between Src activity and boundary translocation; (**J**) spatial CC between Src activity and boundary translocation before and after removing the PDMS membrane.

Based on the calculated boundary correspondence in time, the ECFP/FRET ratio and the translocation can be quantified and visualized as two-dimensional functions of edge location and time (Fig. 4). Therefore, the spatiotemporal maps of ECFP/FRET ratio and boundary translocation in a representative polarized cell were constructed and shown in Fig. 4A,D respectively. The ECFP/FRET ratio image displayed different spatial pattern before (from -7 to 0 min) and after (from 0 to 43 min) the cell was released. Remarkably, the ECFP/FRET ratio of the Src biosensor (representing Src kinase activity) decreased significantly after release at the cell protrusion front but not at the cell rear. This result was further confirmed by the time courses and line scans of the Src ECFP/FRET ratio shown in Fig. 4B,C respectively. The boundary translocation map, time course and the line scan plots of this cell all show that the cell polarized and moved forward notably (~30 μm) at the protrusion front within 10 min after release (Fig. 4D–F), indicating that constraint release remarkably reduced Src kinase activity locally in the protrusive regions of a polarized cell. This unexpected result suggests a novel model that Src activity can be down-regulated at the protrusive front, possibly allowing the activation of the RhoA/mDia signals at the very edge of cell boundary to initiate cell protrusion^16^,^17^,^18^.

To quantitatively evaluate the correlation between Src activity and cell boundary translocation, the temporal and spatial cross-correlation (CC) functions between the Src ECFP/FRET ratio and boundary translocation maps were computed (Fig. 4G,I, Materials and Methods). The cross-correlation functions were normalized to the maximal change of Src activity and translocation among all edge locations to highlight the subcellular regions with significant changes in Src activity and edge translocations. The Src-translocation temporal CC functions were calculated for each sampling window and then aligned along the cell edge to visualize the two-dimensional Src-translocation temporal CC map (Fig. 4G). Since Src activity and cell boundary translocation were negatively coordinated (Src activity decreased while boundary translocation increased), the value of time at the minimum in the 2D temporal CC map (blue color) was expected to represent the time lag between Src down-regulation and cell protrusion. The temporal CC map had a minimal value with a positive time lag (~10 minutes), indicating that Src activity was down-regulated before the start of cell protrusion, which was apparent in a representative protrusive region as shown in the line scan plots (Fig. 4G,H). Similarly, the Src-translocation spatial CC function was calculated from the Src and translocation line scans (with circular extensions) at each time step, normalized to the maximal spatial variation of Src/translocation among all time steps, to highlight the time steps with large subcellular variability (Materials and Methods). Then the spatial CC functions were aligned along time steps to visualize the two-dimensional Src-translocation spatial CC map (Fig. 4I). The spatial CC function had a minimal value (blue color) with a zero shift, confirming that Src down-regulation and cell protrusion occurred at the same regions near cell edge (Fig. 4I,J). The temporal and spatial cross-correlation analysis procedures are detailed and further validated in the Materials and Methods, and Supplementary Information sections (Supplementary Fig. 3).

**Figure 5:**
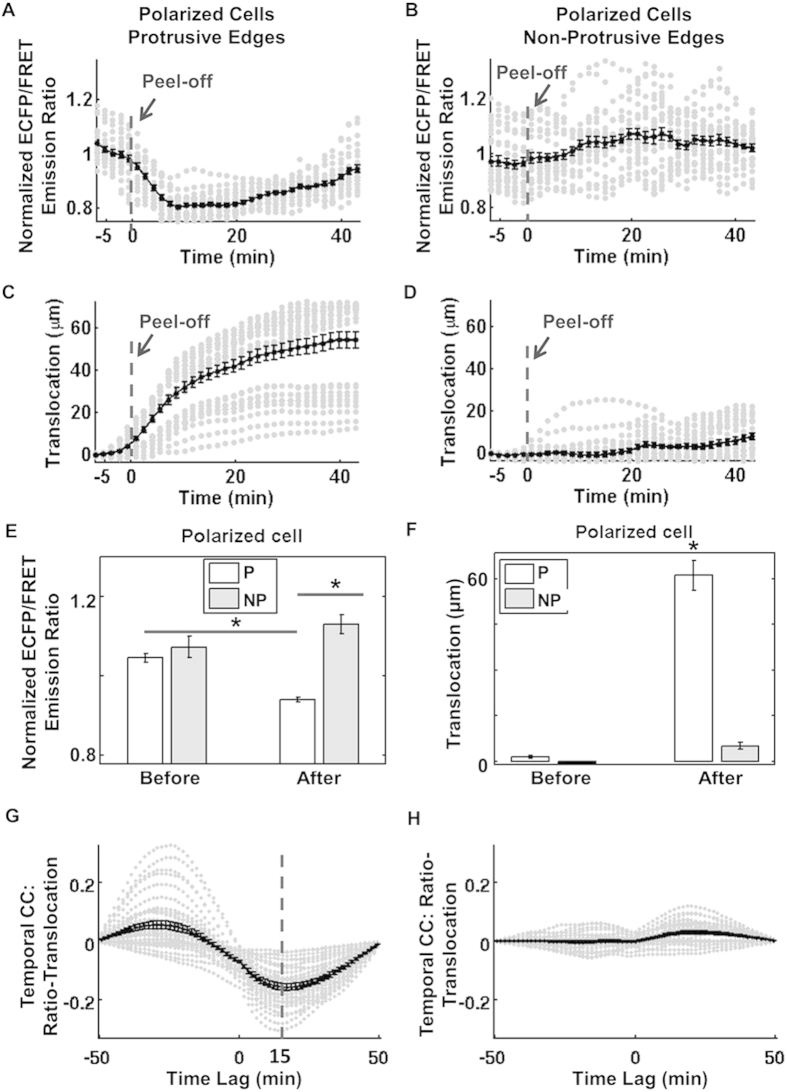
The correlation of Src activity and boundary translocation at the edge of polarized cells. The time courses of normalized ECFP/FRET emission ratio sampled at (**A**) the protrusive edge, or (**B**) non-protrusive edge, of multiple cells; the time courses of translocation sampled at (**C**) the protrusive edge, or (**D**) non-protrusive edge, of multiple cells. (**E**) The maximal ECFP/FRET ratio at the protrusive (P) and non-protrusive (NP) edges are compared before and after peeling-off the PDMS gel membrane; (**F**) The translocation of cell boundary at the protrusive (P) and non-protrusive (NP) edges are compared before and after peeling-off the PDMS gel membrane. The temporal CC at (**G**) the protrusive edge, or (**H**) non-protrusive edge, of multiple cells. Grey dots-data; black solid lines with dots-the average curve. *indicates significant difference by t-test, two tailed, number of cells = 3, number of samples = 40, p-value < 0.05.

The time courses of Src ECFP/FRET ratio and boundary protrusion in the protrusive regions or non-protrusive regions of all the polarized cells were then specifically focused, collected and plotted (Fig. 5A–D). The mean and variance values of the normalized Src activity and boundary translocation were calculated using bootstrapping statistical method with 5000 bootstraps (Fig. 5A–D). Statistical comparison indicated that the Src activity was indeed significantly down-regulated in the protrusive regions of polarized cells, while it was slightly enhanced in the non-protrusive regions of polarized cells (Fig. 5E). As shown in Fig. 5F, it is confirmed that the polarized cells notably moved forward in the protrusive regions, but not in the non-protrusive regions. Similarly, the temporal Src-translocation curves were also collected and plotted for the protrusive regions or non-protrusive regions of all the polarized cells (Fig. 5G,H). The mean of the negative minima among the temporal CC curves (representing the time lag between Src activity and boundary translocation) was about 15 minutes (Fig. 5G), suggesting that the cell membrane protrusion reached the maximal translocation 15 min after Src down-regulation. In comparison, the temporal Src-translocation CC curves had no significant negative minima in the non-protrusive regions of the polarized cells (Fig. 5H), indicating no correlation between the change of Src activity and the movement of cell edge in these regions. Control experiments in cells pre-treated with Src inhibitor PP1 showed that the spatiotemporal pattern of Src kinase activity is inhibited, confirming the specificity of our biosensor results (Supplementary Fig. 4). The cell was able to produce membrane protrusions, suggesting that Src kinase activation is disposable for membrane protrusion in cells released from mechanical constraint (Supplementary Fig. 4).

### In non-polarized cells, Src activity and protrusion were not well-coordinated

**Figure 6:**
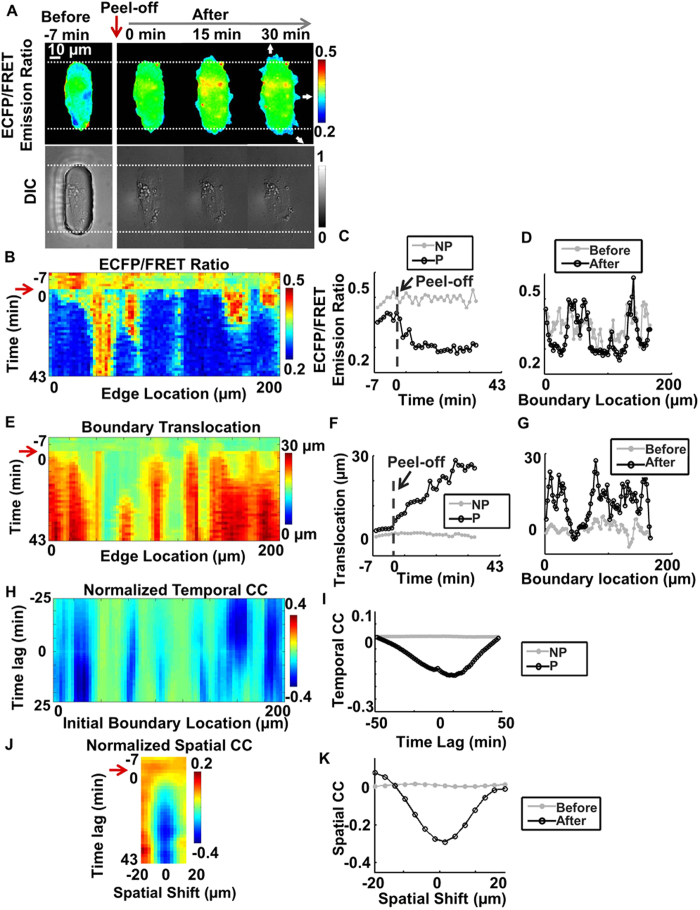
Quantify the dynamic Src activity and membrane protrusion at the edge of a representative non-polarized cell. (**A**) The ECFP/FRET ratio images of Src biosensor at the initiation in a representative non-protrusive cells upon constraint-release (top panels). Lower panels show the corresponding DIC images. (**B**) The spatiotemporal map of ECFP/FRET ratio at cell edge; (**C**) The time courses of Src FRET ratio in a representative sampling window at the protrusive edge (P) and non-protrusive edge (NP); (**D**) line scans of Src FRET ratio along the cell edge before and after removing the gel membrane; (**E**) The spatiotemporal map of cell boundary translocation; (**F**) The representative time courses of edge translocation at the protrusive (P) and non-protrusive (NP) edges; (**G**) The line scans of cell edge translocation before and after removing the PDMS membrane; (**H**) The temporal cross-correlation (CC) map between Src activity and cell edge translocation; (**I**) Temporal CC between Src activity and boundary translocation in two representative sampling windows (P-region and NP-region); (**J**) Spatial CC between Src activity and boundary translocation; (**K**) The spatial CC between Src activity and boundary translocation before and after removing the PDMS membrane.

Based on our observation, about half of the cells polarized after the release of constraint when the restrictive pattern was peeled off, while the other cells expanded without significant polarity, as shown in Fig. 6A. Therefore, the cells were categorized into the polarized or the non-polarized group according to the observed number of protrusive regions in each cell (Materials and Methods, Supplementary Fig. 2). Polarized cells had one significantly protrusive region while non-polarized ones had more than one protrusive region (Supplementary Fig. 2). In non-polarized cells, the Src ECFP/FRET ratio also decreased in multiple protrusive regions at cell membrane (Fig. 6A, Supplementary Fig. 5 and Supplementary Video 2). The Src ECFP/FRET ratio map, the translocation map, and the line scan plots of a representative non-polarized cell also confirmed this observation (Fig. 6A–G). The initial protrusion phase is similar to membrane blebbing as the cell probes the surrounding environment, which does not necessary lead to polarization and migration of the cell. Although non-polarized, the cell expanded up to 30 μm in the localized protrusive regions (after ~43 mins) (Fig. 6E,F), which was similar to the polarized cell. However, the temporal Src-translocation CC map of the non-polarized cell failed to consistently show negative minima, indicating that Src de-activation was not coordinated with membrane protrusion in these cells (Fig. 6H,I). In addition, the spatial Src-translocation CC map of the non-polarized cells had negative minima with a zero shift, indicating that Src down-regulation and membrane protrusion were also spatially aligned in the cell (Fig. 6J,K).

**Figure 7:**
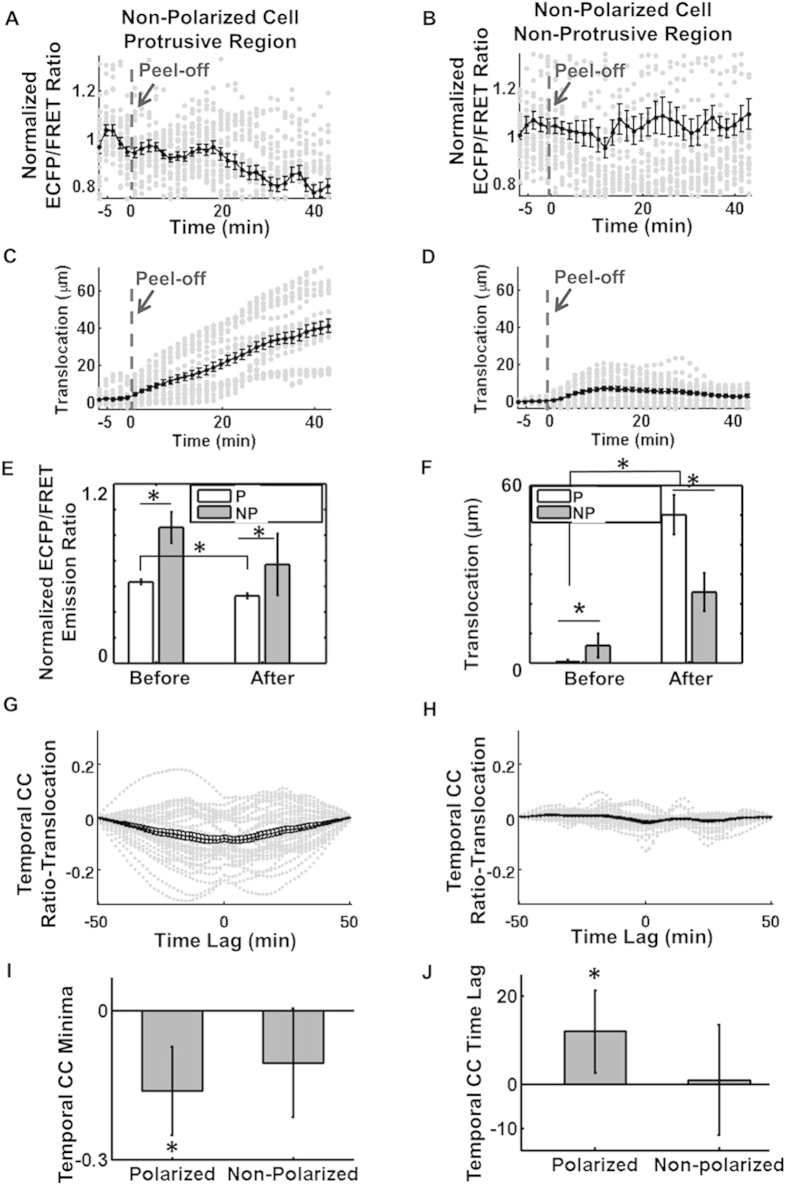
The correlation of Src activity and boundary translocation at the edge of non-polarized cells. The time courses of normalized ECFP/FRET emission ratio sampled at (**A**) the protrusive edge, or (**B**) non-protrusive edge, of multiple cells; the time course of translocation sampled at (**C**) the protrusive edge, or (**D**) non-protrusive edge, of multiple cells. (**E**) The line scans of ECFP/FRET ratio at the protrusive (P) and non-protrusive (NP) edges are compared before and after peeling-off the gel membrane; (**F**) The line scans of boundary translocation at the protrusive (P) and non-protrusive (NP) edges are compared before and after peeling-off the gel membrane. The temporal CC at (**G**) the protrusive edge, or (**H**) non-protrusive edge, of multiple cells. Grey dots-data; black solid lines with dots-the average curve. (**I**) Statistical comparison of the minimal values on the temporal CC curve between Src activity and cell boundary translocation; (**J**) Statistical comparison of the time lag values on the temporal CC between Src activity and cell boundary translocation. *indicates significant difference by T-test, one-tailed, number of cells = 3, number of samples = 40, p-value < 0.05.

As shown in Fig. 7, statistical results from multiple non-polarized cells confirmed the decrease of Src ECFP/FRET ratio, as well as significant expansion of the cell membrane in protrusive regions of the non-polarized cells with multiple protrusive regions. These results confirmed Src down-regulation at the multiple protrusive regions of the non-polarized cells, but not at the non-protrusive regions in the same cells (Fig. 7A–F). In contrast to the Src-protrusion coordination demonstrated in polarized cells, when the temporal Src-translocation curves were collected and plotted for the protrusive regions of the non-polarized cells (Fig. 7G), the mean of the negative minima of the temporal CC curves (representing the time lag between Src activity and boundary translocation) was significantly smaller than that in the polarized cells and not significantly different from zero. The time lags of the negative minima of the temporal CC curves were significantly shorter than those in the protrusive regions of the polarized cells and not significantly different from zero (Fig. 7I,J). These results suggest that in the protrusive regions of non-polarized cells, the observed Src deactivation and membrane protrusion were not coordinated, and that there was no consistent time lag between Src down-regulation and membrane protrusion.

## Discussion

The micro-fabrication technology was integrated with FRET-based biosensors and automated image analysis to build an advanced imaging system in this paper. With the peeling-off of the thin layer of micropatterned PDMS gel membrane during imaging, the cell was triggered to expand and thus allowed us to investigate molecular regulations at the initiation of cell protrusion and migration. The FRET-based Src biosensor was utilized to visualize the change of Src kinases activity in the initial phase of migration. After imaging data acquisition, the level set method was employed to track the spatiotemporal dynamic change of Src kinases along cell boundary. Cross-correlation analysis and statistical inference were used to evaluate the correlation between Src kinase activity and the protrusion dynamics. Thus, the integrated imaging system allowed the investigation of the spatiotemporal coordination between molecular signals and biophysical membrane protrusion during endothelial cell polarization, expansion, and migration.

Here we study the dynamic coordination between Src kinase activity and membrane protrusion when a physical constraint is removed to activate the cell. Our conclusion is also in the context of this changing microenvironment, which is similar to the scenario when endothelial cells bud and extend into space of matrix environment devoid of cells during angiogenesis, or when a tumor cell escapes a stiff microenvironment during metastasis. Our finding that the Src kinase activity was down-regulated at the protrusion front upon constraint release is unexpected and new, since Src activity had been thought to facilitate actin polymerization and cell migration according to classic models^12^,^13^,^46^. Because of the changing microenvironment, the regulation mechanism between Src kinase and protrusion can be different from that in a freely migrating cell. It is likely that the release of micro-pattern removed the compressive force from the constraint on the cell and disrupted the mechanical balance across the plasma membrane, which is different from the random and spontaneous protrusion processes initiated by a freely migrating cell^46^.

Our analysis results indicate that the regulations of Src activity and membrane protrusion are different in polarized and non-polarized cells, although both groups displayed a similar decrease of Src activity in the protrusive regions. In the protrusive regions of the polarized cells, the protrusive events are well-coordinated in space and time after constraint release, with Src activity decreasing for ~15 minutes before the membrane started to protrude in a relatively uniform direction (Figs 4 and 5). As a result, constraint release is expected to induce a directional cell migration with spatiotemporal coordinated Src down-regulation in polarized cells. In non-polarized cells, the protrusive events are poorly coordinated in space and time and the cells exhibited multiple protrusion fronts pointing to different directions after constraint release (Figs 6 and 7), with Src activity decreasing at these regions locally. However, the decrease of Src activity was only spatially co-localized with the protrusion regions in these cells, but was not significantly or consistently earlier than cell protrusion in time. As a result, the cells could not develop a polarized morphology and will not migrate with a clear direction. Therefore, constraint release triggered spatiotemporal coordinated Src down-regulation and membrane protrusion in polarized cells but not in non-polarized cells.

Accordingly, we propose two different mechanistic models: (1) The mechanical relief at the edge and the imbalance can be sensed by the cell and induce rapid actin de-polymerization. Src activity is subsequently down-regulated in these regions^47^. Src de-activation may allow the up-regulation of the RhoA/mDia pathway at the very edge of cell boundary to promote protrusions^12^,^14^. In this case, Src de-activation leads to membrane protrusion, as observed in both polarized and non-polarized cells. (2) The mechanical imbalance triggered membrane protrusion at the subcellular regions under intracellular compressive force (Figs 4 and 7). It is possible that the newly relieved constraint can promote the extension of plasma membrane, which lacks actin network and therefore cannot transport and support Src activity. As a result, membrane protrusion leads to Src de-activation, as observed in the non-polarized cells.

In summary, the integrative system of micro-pattern, FRET imaging and computational analysis allows the novel discovery of spatiotemporally coordinated Src de-activation and membrane protrusion. The results suggest that the constraint-release-induced membrane protrusion in polarized cells do not require Src activation. Therefore, our advanced imaging and analysis system can be applied in the visualization and tracking of these complex and dynamic cellular processes in precisely engineered microenvironment, enabling novel discoveries with important implications in cellular and molecular functions.

## Materials and Methods

This integrative live-cell imaging and analysis system contains three major components: (1) Micro-environment: the peeling-off type of micro-pattern is made by PDMS gel membrane (with wells) which can be peeled off during imaging to release the compressive force at the cells in the well and thus stimulate cell protrusion and migration. (2) Live-cell imaging: genetically-encoded KRas-Src biosensor which enables the imaging and visualization of Src kinase activity during cell protrusion^13^,^30^; (3) Automated image analysis software developed to examine the spatiotemporal relationship between Src activity and cell membrane protrusion upon constraint release.

### The construction of micro-pattern

The restrictive micro-pattern was constructed with a thin PDMS gel membrane (about 40 μm in thickness) engineered by soft lithography via four main steps (Fig. 1A): (1) Before attaching the gel membrane, the extracellular matrix (ECM) protein, Fibronectin (FN), was coated on the surface of glass bottom dishes (Cell E&G) to facilitate cell attachment (10 ng/μl, incubated at 37 °C for 2 hr). (2) A thin layer of PDMS gel membrane with micro-pattern of elliptic shaped wells (20 μm × 80 μm) was created using soft lithography technique^48^ to restrain the cells. This PDMS gel membrane was kept in a 60 mg/ml comb polymer solution with 8:2 (vol:vol) mixture of ethanol and deionized water for 10 s (to prevent cell adhesive ligands binding on the undesired regions of micro-pattern) before attached to FN-coated glass surface. (3) Then a single cell was seeded inside each well of the micro-pattern. (4) During imaging, the thin PDMS gel membrane was peeled off with a pair of tweezers and the compressive force was released, which stimulated the cell to protrude onto the cover glass.

### Live cell imaging

The ECFP/FRET emission ratio of the Src biosensor was used to represent the Src kinase activity. The biosensor was previously developed in our lab and described in various publications (Fig. 1B)^13^,^30^,^31^. The HUVECs were maintained in Dulbecco’s Modified Eagle Medium (DMEM) supplemented with 10% fetal bovine serum (FBS), sodium pyruvate (1 mM), penicillin (1 U/ml), and L-glutamine (2 mM). The HUVECs and cell culture reagents were purchased from ATCC. Cells were cultured in a humidified 5% CO_2_ (and 95% air) incubator at 37 °C. Adenovirus with genetically encoded Src biosensors was used to infect the HUVECs 48 hours before imaging to yield an optimal level of FRET emission light.

Before imaging, the cells were passed to the wells of the micro-patterned membrane and incubated for 2 hours. A CO_2_ air chamber was integrated on the top of this platform to provide a cell culture environment (humidified 5% CO_2_ air) to keep the PH value stable during imaging. The images were collected from a Nikon microscope with a charge-coupled device (CCD) camera. The ECFP/FRET ratio images were generated by our image analysis and visualization software package, *fluocell* (hosted at Google Code), and then subjected to quantification and analysis within MATLAB (Mathworks).

### Imaging analysis methods

Our image analysis and visualization software package *fluocell* was developed to track the cell boundary evolution and detect the spatiotemporal change of Src biosensor activity along the boundary. The overall flow chart of the software program includes cell boundary detection, boundary evolution, time course quantification and cross-correlation analysis (Fig. 2).

To detect the cell edge, the k-means clustering method was used to differentiate the different intensity layers of the Src biosensor. It is relatively robust to imaging-noise and uneven-background. For cell boundary evolution, we adapted the level set method (LSM) developed previously by Machacek *et al.*^14^,^42^. The level set method is an approach for tracking the evolution of complex boundaries, which is briefly described here.

The following equation defines the location of cell boundary at a certain time:


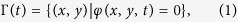


where Γ(*t*) represents the cell boundary at time *t*; the point (*x*, *y*) is located at the cell boundary; *φ*(*x*, *y*, *t*) is the level set function and it represents the signed distance from location (*x*, *y*) to the boundary Γ(*t*) at time *t*. Equation (1) illustrates that the cell boundary Γ(*t*) can be estimated at the zero level of the level set function *φ*(*x*, *y*, *t*) at any time *t*. To simplify calculation, we assume that the cell boundary moves toward its normal direction, and the cell boundary evolution thus can be described as a Hamilton-Jacobi equation:


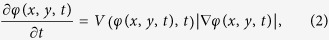


where *V*(*φ*(*x*, *y*, *t*), *t*) is the speed function of level set function *φ*(*x*, *y*, *t*) at time *t*; ∇ represents the gradient operator; and | • | denotes the Euclidean norm. Equation (2) depicts that the cell boundary Γ(*T*) can be propagated to its consecutive frame boundary Γ(*T* + 1) using LSM through estimating the intermediate boundaries (Fig. 3A, for details see supplementary materials).

In general, it is a challenge to accurately align the corresponding boundary points between two time frames, assuming normal propagation (Fig. 3). This problem of misalignment is especially severe at the sharp corners of the boundary with large curvatures at the front or rear parts of cell (Fig. 3B,C). One possible solution is to decrease the step length of the level set method during boundary evolution. However, this will result in a significant increase in computation time.

To balance the computational accuracy and calculation time, we only use a reasonable small step length in the level set method to estimate enough intermediate boundaries between each pair of time frames. Then we apply a spline fit method to map all the intermediate boundaries to a list of 1D spline indices. Therefore, the problem of aligning the boundary between successive frames is reduced to aligning 1D spline indices. During the boundary alignment and propagation, several types of boundary misalignment with geometric conflictions are checked and corrected, as shown in Fig. 2C.

To map Src biosensor activity and boundary protrusion along the cell edge, the sampling window on the Src activity map is selected to be about 0.5 μm in depth and 1.25 μm in width along the cell edge. To build the sampling band, we use a distance map (an image which intensity is the Euclidean distance to the cell boundary) to acquire a constant band with 0.5 μm in depth toward the cell center along cell boundary (Fig. 3C). The windows are then used as masks for sampling Src activity and boundary displacement at a given time and location. The sampling procedure in temporal dimension is done as follows: (1) For the Src activity, the sampling window is used to mask the Src ECFP/FRET ratio image and calculate the average ratio value inside the window; (2) The cell boundary displacement is also calculated by averaging the displacement vectors of boundary points at the corners of the windows; (3) This displacement will be further integrated along time to yield the translocation of cell boundary between two time frames. The word “translocation” is used here to describe the *location change* starting from the position before peeling-off the PDMS gel membrane to the current position. Therefore, both the Src biosensor activity (along the cell boundary) and boundary translocation are sampled into a two-dimensional map. Then the spatiotemporal relationship between Src biosensor activity and cell morphological behavior along the boundary can be studied based on the two-dimensional map (Fig. 4).

For each cell, the boundary was divided into protrusive (P) and non-protrusive (NP) regions, and each sampling window was labeled as one type of regions (Supplementary Fig. 2G). P-regions were determined as regions with peak translocation magnitude larger than or equal to 75% of the maximal translocation over all time frames (Supplementary Fig. 2A and 2G). Further, cells were classified into two groups: polarized cells and non-polarized cells (Supplementary Figs 2A–2F). Polarized cells are cells containing a single P-region (or protrusion front) (Supplementary Fig. 2A and 2B). Cells with more than one P-regions typically do not have a unique protrusion front (Supplementary Fig. 2C and 2D).

To investigate the spatial-temporal relationship between Src activity and cell boundary evolution at migration initiation, the cross-correlation (CC) along the temporal and spatial dimensions was calculated between Src and cell boundary translocation, respectively. The temporal cross-correlation is defined by the following equation:


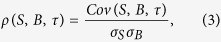


where *S* and *B* represent the time courses of Src activity and cell boundary translocation, respectively; *τ* denotes the temporal difference between two time courses *S*(t) and *B*(t); *Cov*( ) is the covariance of two time courses between Src activity and cell boundary evolution; *σ*_*S*_ and *σ*_*B*_ represent the maximal standard deviations of the centered time courses. And *Cov*( ), *σ*_*S*_ and *σ*_*B*_ are defined as in the following equations:


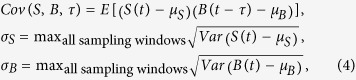


where *E*[ ] represents the expectation value of a random variable; *Var*( ) is the variance; *μ*_*S*_ and *μ*_*B*_ denote the means of the time courses of Src activity and cell boundary translocation, respectively. Equation (3, 4) reflects that the temporal difference *τ* between two time courses can be detected by the normalized cross-correlation and described as peak value of the coefficients. The global normalization with maximum correlation among all sampling window was used to modify the Pearson’s cross-correlation, to highlight the subcellular regions with significant change in Src ECFP/FRET ratio or more cell protrusion. The spatial cross-correlation functions are calculated in a similar fashion to evaluate the spatial shift between the line scans of Src activity and cell protrusion along the whole time course of imaging.

## Additional Information

**How to cite this article**: Zhuo, Y. *et al.* Subcellular and Dynamic Coordination between Src Activity and Cell Protrusion in Microenvironment. *Sci. Rep.* **5**, 12963; doi: 10.1038/srep12963 (2015).

## Supplementary Material

Supplementary materials

Supplementary Movie 1

Supplementary Movie 2
