# Supplementary material for: Subcellular and Dynamic Coordination between Src Activity and Cell Protrusion in Microenvironment
Source: Sci Rep. 2015 Aug 11;5:12963. doi: 10.1038/srep12963 (PMC4531316; doi:10.1038/srep12963)

**Subcellular and Dynamic Coordination between Src Activity and Cell Protrusion in Microenvironment**

Yue Zhuo^1,#^, Tongcheng Qian^1,#^, Yiqian Wu^4^, Jihye Seong^1,6^, Ya Gong^4^, Hongwei Ma^5^, Yingxiao Wang^1,2,3,4,*^, Shaoying Lu^1,4,*^

^1^Department of Bioengineering, ^2^Neuroscience Program, ^3^Center of Biophysics and Computational Biology, Beckman Institute for Advanced Science and Technology, Department of Molecular and Integrative Physiology and, University of Illinois at Urbana-Champaign, Urbana, IL 61801, ^4^Department of Bioengineering, Institute of Engineering in Medicine, University of California, San Diego, La Jolla, CA 92093-0435, ^5^Suzhou Institute of Nano-Tech and Nano-Bionics, Suzhou, China, ^6^Center for Neuro-Medicine, Brain Science Institute, Korea Institute of Science and Technology (KIST), Seoul, South Korea

^#^These authors contributed equally to this work.

### *To whom correspondence should be addressed.

Shaoying Lu, Ph. D.

Department of Bioengineering

University of California, San Diego, La Jolla, CA 92093-0435

Tel: (858) 822-4502

Fax: (858) 822-1160

E-mail: [kalu@eng.ucsd.edu](mailto:kalu@eng.ucsd.edu)

Yingxiao Wang, Ph. D.

Department of Bioengineering, the Institute of Engineering in Medicine,

University of California, San Diego, La Jolla, CA 92093-0435

Tel: (858) 822-4502

Fax: (858) 822-1160

E-mail: [yiw015@eng.ucsd.edu](mailto:yiw015@eng.ucsd.edu)

**Supplementary Materials**

**Computational methods for boundary evolution**

$\mathop{\lim V(\phi_{t},t)}\limits_{\phi_{t}\to\phi_{T+1}} =0,$The speed function of level set for cell boundary evolution can be described as the following equation:

(S1)

where *φ_T+1_* is defined as *φ_T+1_*=*φ*(*x*, *y*, *T*+1) at translocation (*x*, *y*) and time *T*+1. Equation (S1) expresses that the choice of speed function *V* will not only influence the shape of the intermediate boundaries, but also the convergence of the boundaries. In this work, the speed function *V* used is described in [41], and it is shown in equation (S2):

$$V(\phi_{t},t)=\left\{\begin{matrix} (\phi_{T+1}-\phi_{t})(\varepsilon+\kappa),\begin{matrix} & \end{matrix}for(\phi_{T+1}-\phi_{t})\geq0, \\ ar\sinh(\phi_{T+1}-\phi_{t}),\begin{matrix} & \end{matrix}for(\phi_{T+1}-\phi_{t})<0, \end{matrix}\right.$$

(S2)

$\kappa=\frac{\phi_{xx}\phi_{y}^{2}-2\phi_{x}\phi_{y}\phi_{xy}+\phi_{x}^{2}\phi_{yy}}{\left(\phi_{x}^{2}+\phi_{y}^{2}\right)^{\frac{3}{2}}},$where *ar*sinh( ) is the inverse hyperbolic function; the small constant ε is determined by computational grid size. The curvature *κ* of the cell boundary can be estimated by a second order finite difference function as shown in equation (S3):

(S3)

where *φ_x_* denotes the first-order derivative of *φ*(*x*,*y*,*t*) with respect to *x*; *φ_xy_* denotes the second-order derivative of *φ*(*x*,*y*,*t*) taken respect to x and y; and so on.

**Boundary Evolution Implementation**

A classical numerical method, Runge-Kutta method, is chosen to solve the boundary evolution function in equation (2) [41]. The Runge-Kutta methods are a family of iterative methods providing numerical solutions to ordinary differential equations. The forth order Runge-Kutta method (RK4), also known as classic Runge-Kutta method, is the most commonly utilized numerical method.

Let us rephrase equation (2) to an explicit ordinary differential equation, as in equation (S4):

$$\phi'=f(t,\phi),$$

(S4)

$\begin{matrix} \phi_{n+1}=\phi_{n}+\frac{h}{6}(k_{1}+2k_{2}+2k_{3}+k_{4}), \\ t_{n+1}=t_{n}+h, \end{matrix}$where the initial value at time *t_0_* is denoted as *φ*(*t_0_*) = *φ_0_*. Then the numerical solution can be iteratively approximated with RK4, as shown in equation (S5):

(S5)

$\begin{matrix} k_{1}=f(t_{n},\phi_{n}),\begin{matrix} \\ \end{matrix} \\ k_{2}=f(t_{n}+\frac{1}{2}h,\phi_{n}+\frac{1}{2}k_{1}), \\ k_{3}=f(t_{n}+\frac{1}{2}h,\phi_{n}+\frac{1}{2}k_{2}), \\ k_{4}=f(t_{n}+h,\phi_{n}+k_{3}).\begin{matrix} \\ \end{matrix} \end{matrix}$where *h* denotes the time interval; the weighing parameters *k_i_* (*i* = 1 ~ 4) are the slope estimated at the beginning, midpoints (with higher weighting value), or end of the consecutive intervals, and their values are described in equation (S6):

(S6)

Note that the approximation error per step is on the order of *h^5^* and overall error is on the order of *h^4^* (since RK4 is a forth order numerical method).

$r=\sqrt{Tr(D^{T}D)},$In this work, the RK4 is applied with an adaptable time step during iteration computations to preserve uniform distances between intermediate frames for each boundary points, as in [41]. The step size of iteration is determined by the Frobenius norm *r* for the difference between two consecutive level set functions, as shown in the following equation:

(S7)

where *Tr*( ) represents the trace of a matrix; and the difference *D* is defined as in equation (S8):

$$D=\phi_{T+1}-\phi_{t}.$$

(S8)

The threshold *r_0_* of Frobenius norm is selected empirically (i.e. *r_0_* = 5) to preserve the approximation accuracy underneath certain computation time. The iterative computation is terminated when the calculated Frobenius norm *r* is over the threshold *r_0_*. In this work, the number of iterations is typically around 20 between each pair of consecutive cell boundaries. In general, small step size will yield more intermediate boundaries which provide more accurate displacement vectors. However, the price for this accuracy is to spend more computation time.

**Validation of the computational analysis results and conclusion**

To validate the computational analysis results and conclusion, a simulation of migrating cell was generated. As shown in Supplementary Figures 3A-3B, the simulated cell mimics the migration pattern and the change of Src kinase activity observed in HUVECs: (1) For the first 10 minutes, the cell stayed still; (2) Then it started to migrate upward with a uniform speed. (3) The Src activity in the front began to decrease after 10 minutes, which was negatively coordinate with the cell movement. Then our imaging analysis software was applied on this computer-generated cell and the results are depicted in Supplementary Figures 3C-3J. The 2D temporal CC and spatial CC maps resemble those for migration cells in Figures 4G and 4I. The temporal CC map had a negative minimum with a positive time lag (Supplementary Fig. 3G), while the spatial CC map had a negative minimum with a zero-shift within the confidence region of 20 µm from 0 (Supplementary Fig. 3I). The artificial pattern of positive maximal values in the spatial CC map outside of the confidence region was caused by the periodic extension of Src activity and boundary translocation in space along the boundary location, but not an indication of positive correlation between these two signals (Fig. 4I and Supplementary Fig. 3I). So the positive maximal values were not included in the analysis. Therefore, the results confirm that our imaging analysis methods can dynamically detect the Src activity and its temporal-spatial relationship with the cell boundary movement when migration started. The analysis results from simulation with known correlation in the signals also support our biological interpretation of the temporal and spatial Src-translocation CC maps.

**Supplementary Figure Legends**

**Supplementary Figure 1.** The ECFP and FRET intensity images showing the biosensor distribution in the representative polarized cell shown in Figure 1. The biosensor was distributed at the cell membrane before and after the constraint release, with the FRET intensity about 3-5 folds of the ECFP intensity in the cell. **Top panel:** ECFP intensity images; **Lower panel:** FRET intensity images.

**Supplementary Figure 2. Criteria to differentiate between the polarized and non-polarized cells, or between the protrusive and non-protrusive regions. (A)** The smoothed boundary translocation map of a representative polarized cell; **(B)** The line scans of boundary translocation and the number of peaks of this polarized cell (from 13 to 30 mins); **(C)** The smoothed boundary translocation map of one non-polarized cell; **(D)** for the line scans of boundary translocation and the number of peaks of this non-polarized cell (also from 13 to 30 mins); **(E)** The threshold to classify polarized (P) and non-polarized cells (NP) according to the number of peaks in the translocation line scans; **(F)** Statistical comparison of number of peaks between polarized and non-polarized cells; **(G)** The threshold to partition the cell edge into protrusive (P) and non-protrusive (NP) regions based on the histogram of boundary translocation in a polarized cell. * indicates significant difference by t-test, number of cells = 3, p<0.05.

**Supplementary Figure 3. The computer simulated experiment. (A)** The change of Src activity in a simulated cell. **(B)** The designated change of Src activity in time; **(C)** The sampled spatiotemporal map of the Src activity along the cell edge; **(D)** The sampled spatiotemporal map of boundary translocation; **(E)** The time courses of Src activity and boundary translocation in a sampling window; **(F)** The line scans of Src activity and boundary translocation after the cell started moving; **(G)** The temporal CC map between Src activity and boundary translocation; **(H)** The temporal Src-translocation CC curve within a sampling window of the protrusive region; **(I)** The spatial CC map between Src activity and boundary translocation along cell edge and the time dimension. The region of confidence is indicated between the locations of two arrows. **(J)** The spatial Src-translocation CC curve in a representative time frame after the cell started moving.

**Supplementary Figure 4.** Src-inhibition PP1 (1 µM, 1h pre-treatment) removed the observed spatiotemporal pattern of Src kinase activity in cells released from constraint. The PP1-treated HUVEC was released from the micropattern constraint. **Top panels** show the ECFP/FRET ratio images of the cell before and after release; **lower panels** show the FRET intensity images of the same cell.

**Supplementary Figure 5.** The ECFP and FRET intensity images showing the biosensor distribution in the representative non-polarized cell shown in Figure 6. The biosensor was distributed at the cell membrane before and after the constraint release, with the FRET intensity about 3-5 folds of the ECFP intensity in the cell. **Top panel:** ECFP intensity images; **Lower panel:** FRET intensity images.

**Supplementary Video Legends**

**Supplementary Video 1.** The spatiotemporal dynamic change of Src kinase activity in a polarized HUVEC cell stimulated by constraint release. **Top Left:** The Src ECFP/FRET ratio representing the Src kinase activity in the cell before and after constraint release; **Top right:** The DIC image showing the localization of the cell; **Bottom Left:** the ECFP intensity images; **Bottom Right:** the FRET intensity images showing the localization of the biosensor in the cell. This cell is also shown in Figure 1.

**Supplementary Video 2.** The spatiotemporal dynamic change of Src kinase activity in a non-polarized HUVEC cell stimulated by constraint release. **Top Left:** The Src ECFP/FRET ratio representing the Src kinase activity in the cell before and after constraint release; **Top right:** The DIC image showing the localization of the cell; **Bottom Left:** the ECFP intensity images; **Bottom Right:** the FRET intensity images showing the localization of the biosensor in the cell. This cell is also shown in Figure 6.


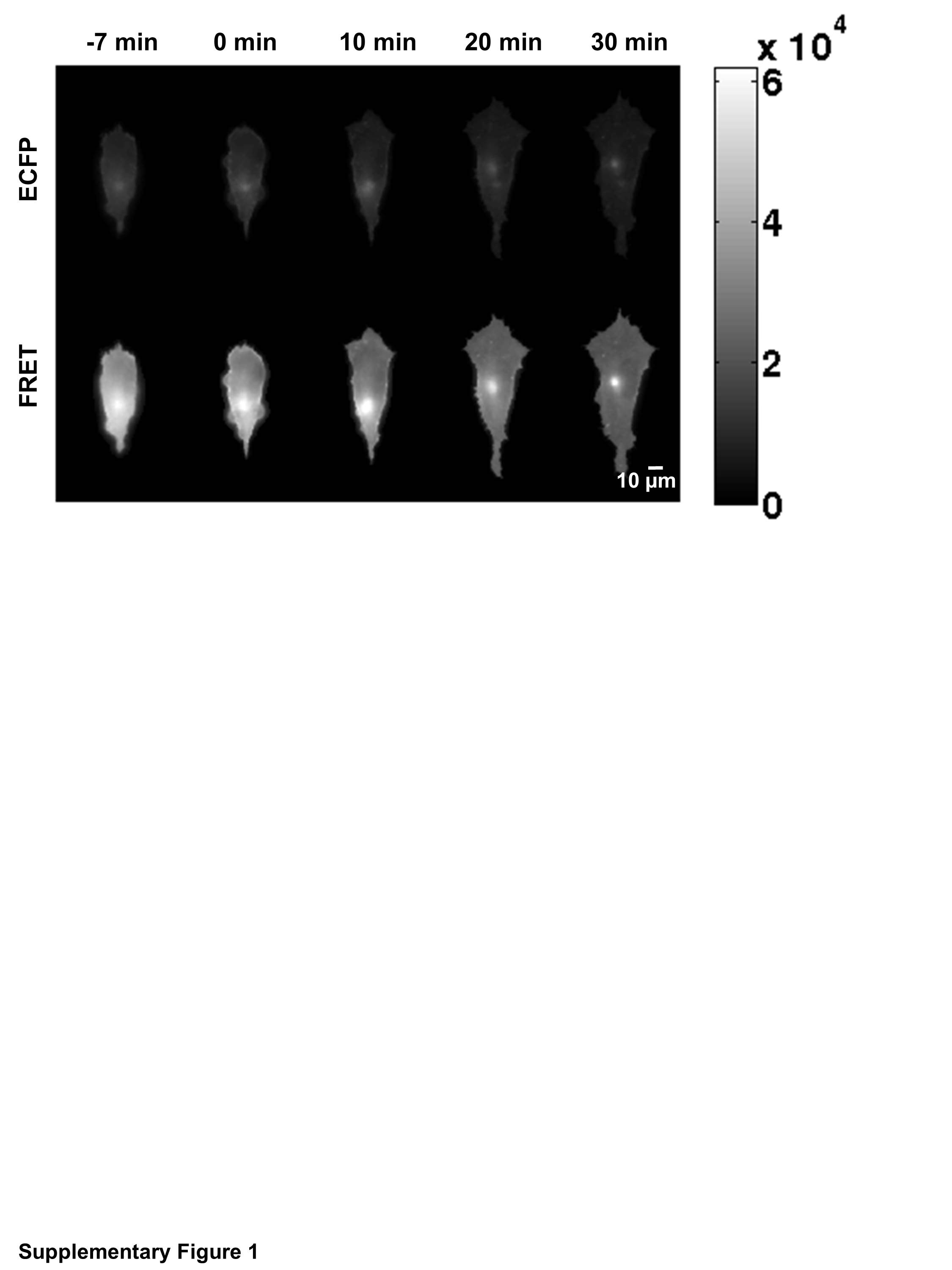

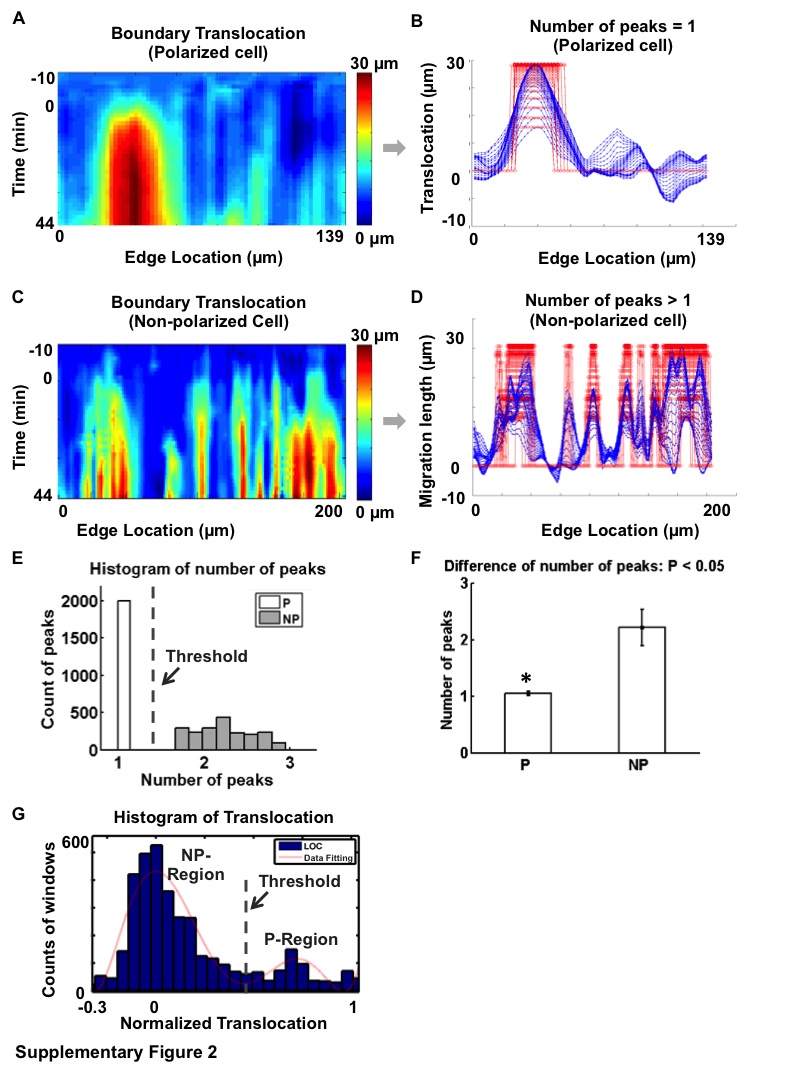

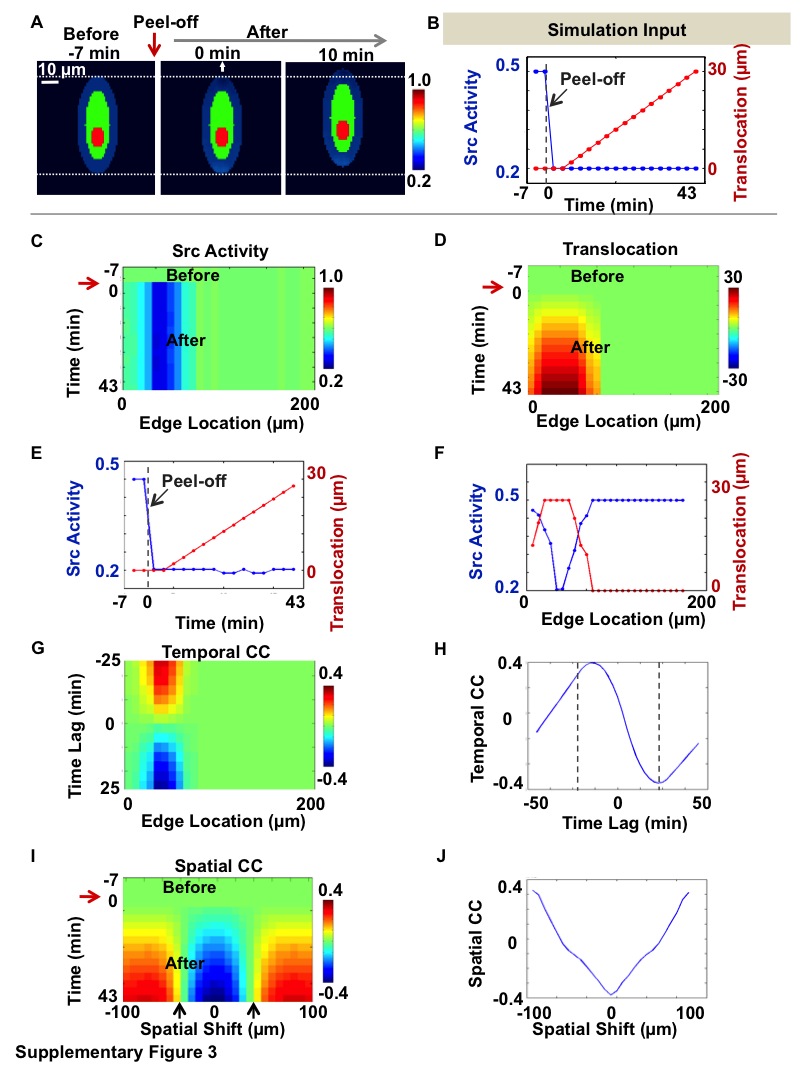

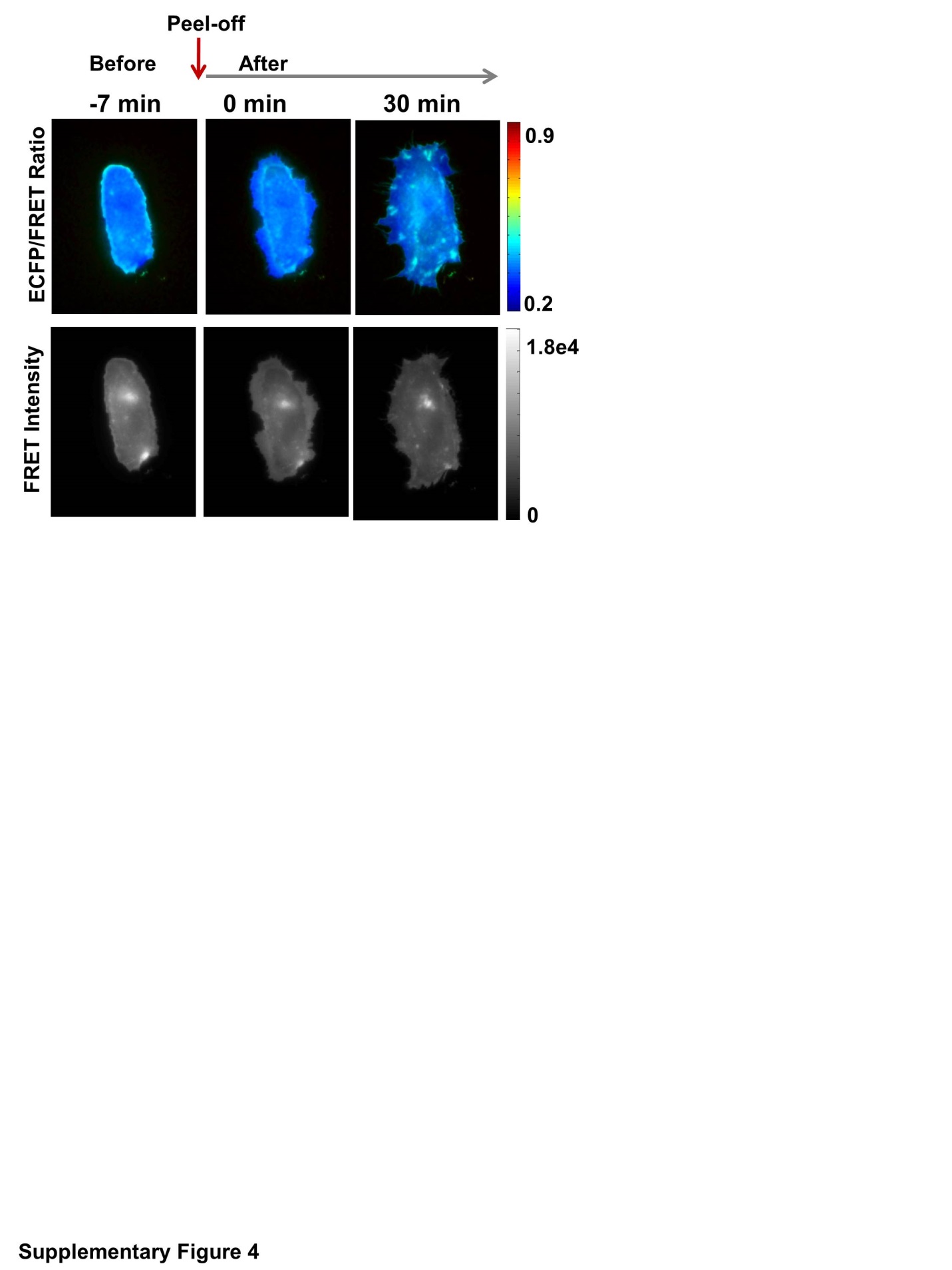

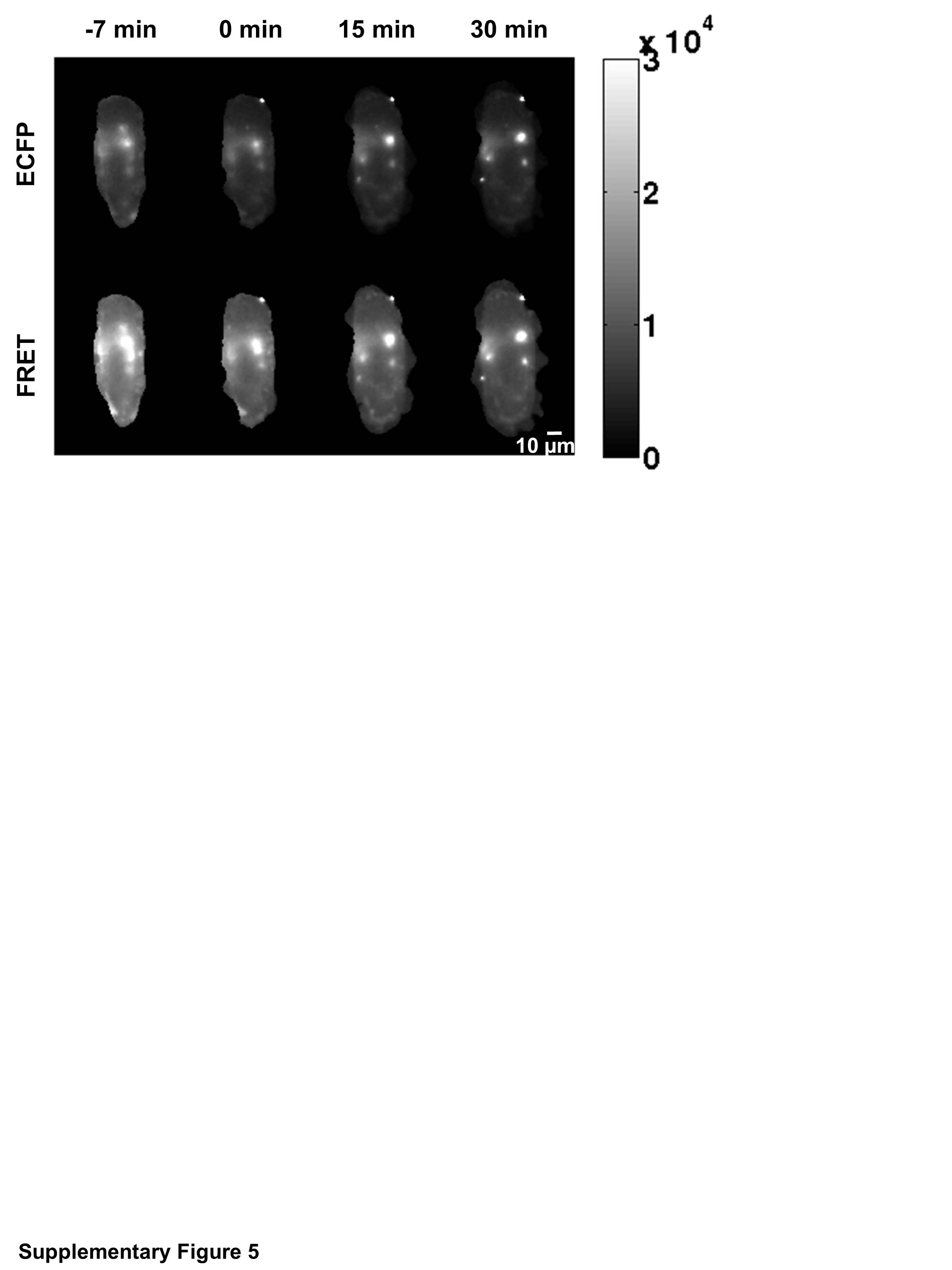

Supplement: Supplementary materials [file srep12963-s1.doc]
